# Genomic Characterization and Expression of Juvenile Hormone Esterase-Like Carboxylesterase Genes in Pacific White Shrimp, *Litopenaeus vannamei*

**DOI:** 10.3390/ijms21155444

**Published:** 2020-07-30

**Authors:** Xiaoxi Zhang, Jianbo Yuan, Xiaojun Zhang, Jianhai Xiang, Fuhua Li

**Affiliations:** 1 CAS Key Laboratory of Experimental Marine Biology, Institute of Oceanology, Chinese Academy of Sciences, Qingdao 266071, China; zhangxiaoxi@qdio.ac.cn (X.Z.); jhxiang@qdio.ac.cn (J.X.); fhli@qdio.ac.cn (F.L.); 2 Laboratory for Marine Biology and Biotechnology, Qingdao National Laboratory for Marine Science and Technology, Qingdao 266237, China; 3 Center for Ocean Mega-Science, Chinese Academy of Sciences, Qingdao 266071, China; 4 University of Chinese Academy of Sciences, Beijing 100049, China

**Keywords:** juvenile hormone esterase-like carboxylesterase, development, molting, penaeid shrimp

## Abstract

The sesquiterpenoid methyl farnesoate (MF), a juvenile hormone (JH) analog, plays important roles in many physiological processes of crustaceans, such as morphogenesis, molting and reproduction. Juvenile hormone esterase-like (JHE-like) carboxylesterase (CXE) is a key enzyme in MF degradation, playing a significant role in regulating MF titer. However, its function is barely known in shrimp. In this study, a total of 21 JHE-like CXEs (*LvCXE*s) were characterized in Pacific white shrimp *Litopenaeus vannamei*, based on the full genome and multi-transcriptomic data. *LvCXE* has a conserved triplet catalytic site (Ser-Glu-His) and a characteristic GxSxG motif. Most *LvCXE*s were highly expressed in the hepatopancreas, which was the main site for MF degradation. *LvCXE*s containing a GESAG motif showed a specific expansion in the *L. vannamei* genome. Those GESAG-containing *LvCXE*s presented differential expressions at different larvae stages and different molting stages of *L. vannamei*, which suggested their potential functions in development and molting. Additionally, when the transcription level of CXEs was inhibited, it could lead to failed molt and death of *L. vannamei*. When we further detected the expression levels of the key ecdysone responsive transcription factors including *LvE75*, *LvBr-C*, *LvHr3* and *LvFtz-f1* after the CXE inhibitor was injected into *L. vannamei*, they all showed apparent down-regulation. These results suggested that the expansion of *LvCXE*s in the *L. vannamei* genome should contribute to the regulation of metamorphosis at larvae stages and frequent molting during the growth of *L. vannamei*.

## 1. Introduction

Carboxylesterase is a superfamily of multifunctional enzyme which ubiquitously exists in animals, plants and microbes [1,2]. Based on sequence similarity and substrate specificity, carboxylesterases can be divided into eight subfamilies—α-esterases (Ae), β-esterases (Be), juvenile hormone esterases (JHE), gliotactins (Gli), acetylcholinesterases (AChE), neurotactins (Nrt), neuroligins (Nrl) and glutactins (Glt) [3]. Among them, JHE is considered to be the predominant enzyme involved in degradation of juvenile hormone (JH), which is a group of sesquiterpenoid compounds with pleiotropic functions in development, metamorphosis, molting, growth, reproduction and pheromone biosynthesis of insects [4,5]. JHE converts JH to JH acid and then regulates JH titer at appropriate levels, and thereby directs development and metamorphosis in insects [6,7]. The increase of JHE activity at late developmental stage is one of the important biochemical events that leads to pupation [8,9]. Moreover, inhibition of JHE activity maintained JH titer, led to abnormally large larvae and delayed metamorphosis [10,11].

JH has never been identified in crustaceans, and the only JH-like compound, named as methyl farnesoate (MF), is an unepoxidated form of insect JH Ш [12]. MF is produced in the mandibular organs (MOs) of crustaceans and possesses similar functions with insect JH, which is involved in many crucial physiological processes such as morphogenesis, molting, growth and reproduction etc. [12,13,14,15]. For example, exposure of lobster larvae to MF increased the duration of their development [16], and addition of MF to the feed of freshwater prawn retarded the growth and development at late larval stages [17]. Meanwhile, the role of MF during the molting cycle was uncertain. For example, MF can accelerate molting in crayfish *Cherax quandricarinatus* [18] and *Procambarus clarkii* [15], but causes molt-related mortality and delay the molting process of Artemia larvae [19]. The regulation of the molting process is complicated, and it experiences the following processes. The titer of MF and ecdysone (molting hormone, a steroid compound derived from cholesterol) presents periodic fluctuation in hemolymph of adult crustaceans [20,21,22]. In detail, MF and ecdysone titer increases gradually at the early pre-molt stage and reaches a maximum near molting, then decreases rapidly at the late pre-molt stage. It leads to large scale synthesis of ecdysone in Y organ at the pre-molt stage owing to the negative feedback [23]. Meanwhile, the expressions of ecdysone receptor (EcR) and retinoid X receptor (RXR), which form a heterodimer to which ecdysone binds, are induced [24]. As early response genes, Broad-complex (Br-C), E74 and E75 are then normally up-regulated by EcR. After that, several early late genes such as hormone receptor 3 (Hr3) and late response genes such as Ftz-f1, are expressed [25]. Finally, the molting process occurs. Therefore, a rapid decrease of both MF and ecdysone titer in hemolymph near molting is the key factor for successful molting [5]. When injecting 20E into crayfish at the late pre-molt stage, the synthesis of ecdysone in Y organ decreased and the normal molting cycle of crayfish delayed [26].

There are many ways to study the function of JHE in insects. For example, gene knockout, RNA interference and inhibitor injection. Among them, OTFP (3-octylthio-1,1,1-trifluoropropan-2-propanone), a slow and tight-binding inhibitor of JHE, was widely used to investigate the function of JHE owing to the fact that it can suppress more than 98% of the JHE activity in insects [27,28]. Furthermore, OTFP had also been used in crustaceans, such as the spider crab *Libinia emarginata* [29]. However, due to the weakness of the experimental platform, the study on the function of juvenile hormone esterase-like (JHE-like) carboxylesterases (CXE) in crustaceans is limited. Like insects, CXE was supposed to degrade MF through ester hydrolysis in crustaceans, and it was supported by the results that the expression of CXE genes was up-regulated by eyestalk ablation or MF treatment in several crustacean species [30,31,32,33]. Nevertheless, they mainly focused on the role of CXE in reproductive development and the innate immune response [30,31,32,33], whereas their functions in early development and the molting process are barely known in crustaceans. The Pacific white shrimp *Litopenaeus vannamei* is one of the most economically important marine aquaculture species in the world (FAO). Additionally, the penaeid shrimp experiences about 50 molts during a lifetime [34], far more than other arthropods [35]. The expansion of many molt-related gene families, such as *Broad-Complex* (Br-C), the crustacean hyperglycemic hormone (CHH) and chitinase family, contributed to frequent molting of *L. vannamei*. However, a previous study has also found that the CXE gene family showed expansion in *L. vannamei* genome through comparative genomic analysis [36], whereas the relationship between the CXE gene family and molting process of *L. vannamei* remains uncertain. In this study, we characterized *LvCXE*s in *L. vannamei* genome comprehensively and conducted phylogenetic analysis on these *LvCXE*s. We then illustrated their expressions at early developmental stages and investigated the relationships between the expansion of the CXE gene family and frequent molting of *L. vannamei*. The OTFP inhibition experiment was performed to validate their functions in the molting process of *L. vannamei*. The results obtained in this study will enable us to better understand the function of CXEs during the early developmental stages and molt cycle of *L. vannamei* and even other crustaceans.

## 2. Results

### 2.1. Characterization of CXE Genes in L.vannamei genome

**Figure 1:**
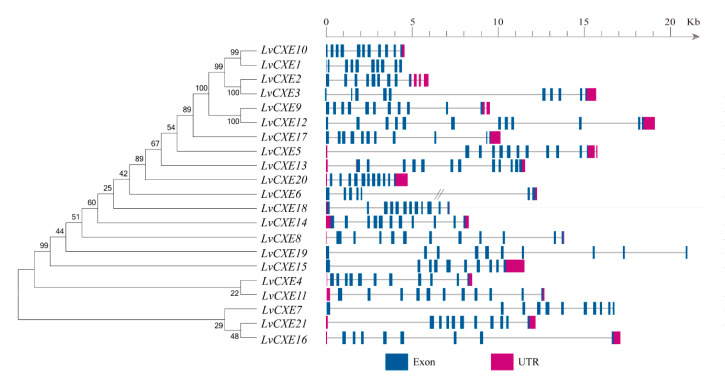
Schematic representation of exon-intron structures of the identified *LvCXE*s. The maximum likelihood (ML) phylogenetic tree on the left was generated using RAxML software with 100 bootstrap replicates. Exons, introns and untranslated regions (UTRs) are shown as blue rectangles, grey lines and rose red rectangles, respectively. Double slash represents a gap of genome scaffold.

Among 51 CXE genes, a total of 21 *LvCXE*s held a complete open reading frame (ORF), which were named as *LvCXE1-LvCXE21*, respectively. The predicted proteins of these *LvCXE*s varied from 488 to 669 amino acid residues. The estimated Mw ranged from 54.3 to 74.5 kDa, with pI of 4.53 to 8.98 (Table 1). The exon numbers of all *LvCXE*s varied from 8 to 13 (Figure 1). *LvCXE2* and *LvCXE3* were distributed in scaffold883, *LvCXE4* and *LvCXE5* located in scaffold962, *LvCXE9* and *LvCXE10* were distributed in scaffold1839. Other *LvCXE*s scattered in *L. vannamei* genome (Table 1).

### 2.2. Structure Analysis of LvCXEs

**Figure 2:**
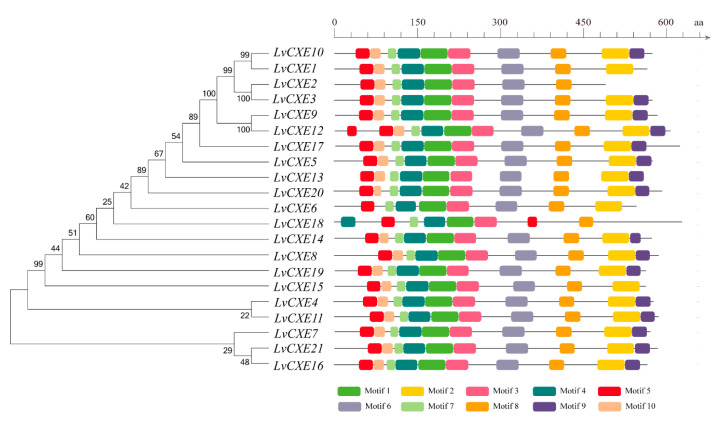
Schematic representation of conserved motifs identified in LvCXEs. The ML phylogenetic tree on the left was generated using RAxML software with 100 bootstrap replicates. Different color boxes represent different types of motifs, and the names of all members are shown on the bottom.

**Figure 3:**
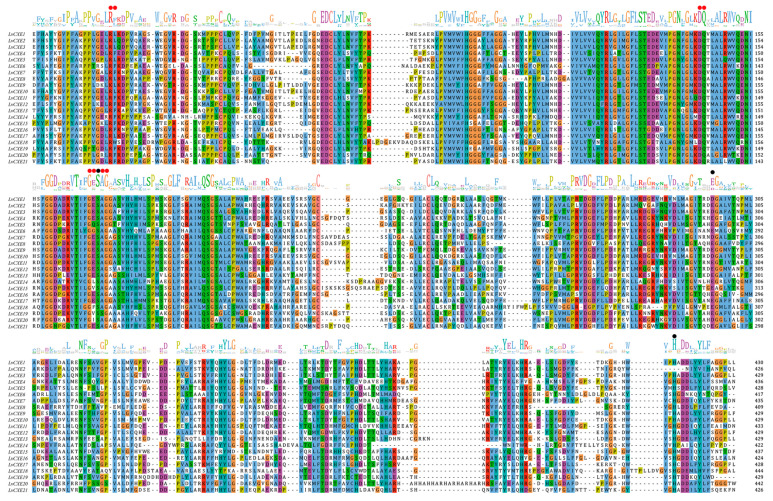
Multiple alignment of amino acid sequences containing conserved motifs and domains of LvCXEs. Black circles indicate the catalytic triad residues (S, E, H) and red circles indicate conserved motifs of carboxylesterases.

A total of 15 of 21 LvCXEs contained ten motifs (Figure 2), indicating that their protein sequences showed high similarity. Among ten motifs, motif 5, 4, 1, 3, 6 contained the RF, GG, DQ, GxSxG, E/D motif, respectively, which were mentioned in Table 2. Except for the incomplete LvCXE12, all LvCXEs were expected to contain a signal peptide sequence (17–26 aa) at the N-terminal (Table 2). The multiple alignment revealed that all LvCXEs had a typical domain organization, including a conserved catalytic triad (Ser-Glu-His) and a carboxylesterase-specific GxSxG motif. A total of 14 LvCXEs contained the GESAG motif, and the other LvCXEs were different. In addition, LvCXEs had RF (arginine, phenylalanine) and DQ (aspartic acid, glutamine) residues at the same position as insect JHEs (Figure 3 and Table 2).

### 2.3. Phylogenetic Analysis

**Figure 4:**
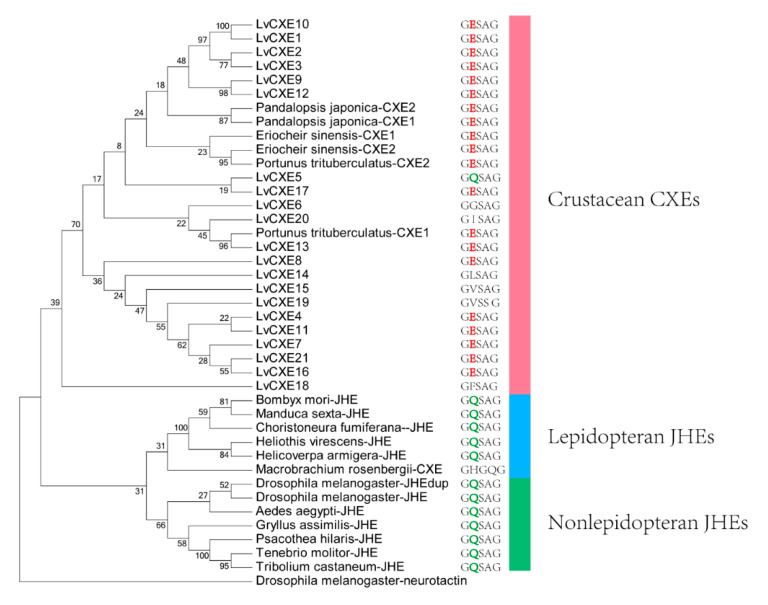
The ML phylogenetic tree of juvenile hormone esterases (JHEs) and CXEs was generated using RAxML software with 100 bootstrap replicates. All deduced amino acid sequences were obtained from GenBank: *Drosophila melanogaster*-neurotactin (NP_730196.1), *Heliothis virescens*-JHE (AAC38822.1), *Helicoverpa armigera*-JHE (AEB77712.1), *Bombyx mori*-JHE (AAR37335.1), *Choristoneura fumiferana*-JHE (AAD34172.1), *Tenebrio molitor*-JHE (AAL41023.1), *Tribolium castaneum*-JHE (BAJ10679.1), *Psacothea hilaris*-JHE (BAE94685.1), *Gryllus assimilis*-JHE (ABQ23214.1), *Aedes aegypti*-JHE (AAEL005200-PA), *D. melanogaster*-JHE (NP_001286476.1), *D. melanogaster*-JHEdup (NP_611085.2), *Manduca sexta*-JHE (AAG42021.2), *Eriocheir sinensis*-CXE1 (A0A1L5JHT6), *E. sinensis*-CXE2 (A0A1L5JHU7), *Portunus trituberculatus*-CXE1 (A0A1I9KY23), *P. trituberculatus*-CXE2 (A0A1I9KJ57), *Pandalopsis japonica*-CXE1 (F5A5Q6), *P. japonica*-CXE2 (F5A5Q7), *Macrobrachium rosenbergii*-CXE (MG910496.1). The neurotactin of *D. melanogaster* was used as an outgroup.

The phylogenetic analysis was conducted to investigate the evolutionary relationship between LvCXEs, insect JHEs and other crustacean CXEs. The results showed that all LvCXEs were clustered within the monophyletic clade of crustacean CXE (Figure 4). Meanwhile, JHE genes of insect were clustered in another monophyletic clade. Additionally, most LvCXEs containing the GESAG motif were closely related to each other in the phylogenetic tree, suggesting that GESAG-containing CXE genes expanded specifically in *L. vannamei*. Furthermore, most of JHE genes of insect contained the GQSAG motif rather than GESAG.

### 2.4. Spatial and Temporal Expression of LvCXEs

**Figure 5:**
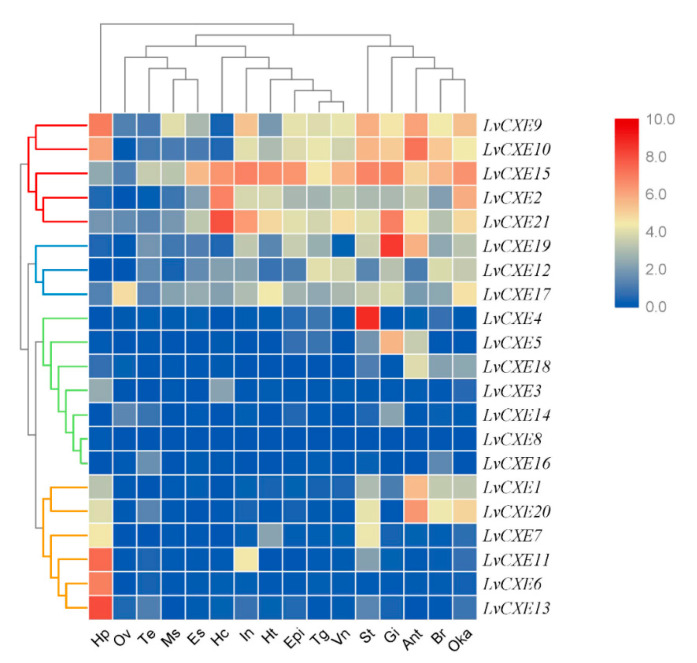
The expression profiles of *LvCXE*s in 16 adult tissues. Log2-transformed expression values were used to create the heat map. Red or blue colors represent the higher or lower relative abundance, respectively. Red, blue, green and yellow clades on the left representemodule 1–4, respectively, which were clustered by hierarchical clustering. *LvCXE*s shared similar expression profiles in each module. The bottom lists adult tissues of *L. vannamei*. (Hc: hemocyte, Ant: antennary gland, Ms: abdominal muscle, In: intestine, Ov: ovary, St: stomach, Oka: lymphoid organ, Gi: gill, Hp: hepatopancreas, Te: testis, Es: eyestalk, Br: brain, Tg: thoracic ganglion, Vn: ventral nerve, Epi: epidermis, Ht: heart).

RNA-seq data was used to analyze the spatial expression patterns of *LvCXE*s in 16 different tissues of the adult *L. vannamei*. The hierarchical clustering analysis showed that the expression patterns of *LvCXE*s can be divided into 4 modules (Figure 5). Module 1, including *LvCXE2*, *LvCXE9*, *LvCXE10*, *LvCXE15* and *LvCXE21*, was found to be expressed in almost all tissues detected, with exception of ovary, testis and muscle. Module 2, including *LvCXE12*, *LvCXE17*, *LvCXE19*, was also expressed in all tissues detected, but the expressions was lower than group 1. Module 3, including *LvCXE3*, *LvCXE4*, *LvCXE5*, *LvCXE8*, *LvCXE14*, *LvCXE16* and *LvCXE18*, showed expression in specific tissues. For example, *LvCXE4*, *LvCXE5* and *LvCXE18* were almost specifically expressed in stomach, gill and antennary gland, respectively. Module 4, including *LvCXE1*, *LvCXE6*, *LvCXE7*, *LvCXE11*, *LvCXE13* and *LvCXE20,* was highly expressed in hepatopancreas.

**Figure 6:**
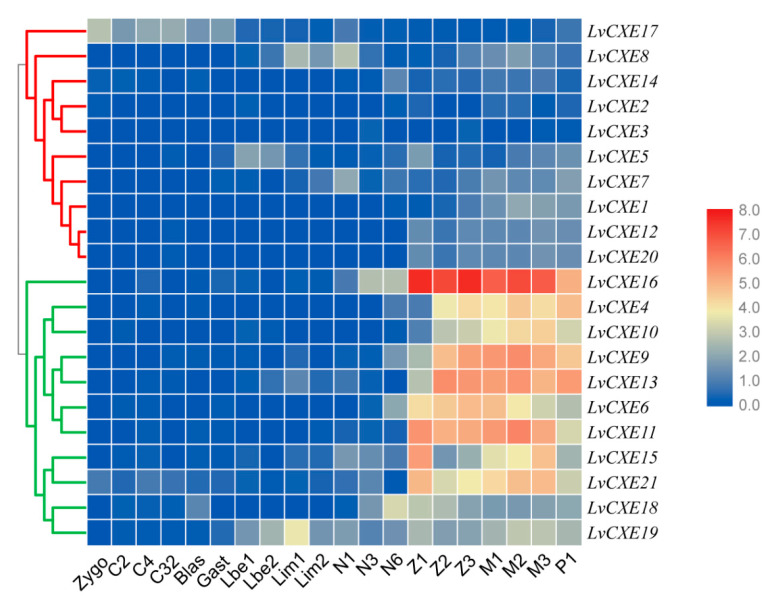
The expression profiles of *LvCXE*s during development of *L. vannamei*. Each rectangle with a white line represents the expression level of corresponding genes (right) in the corresponding stages (bottom). Blue and red colors represent low and high expressions, respectively. Red and green clades on the left represente module 1 and module 2, respectively, which were clustered by hierarchical clustering. *LvCXE*s shared similar expression profiles during the early development in each module. The bottom lists different larval stages of *L. vannamei*, from left to right—zygote, 2 cells (C2), 4 cells (C4), 32 cells (C32), blastula (blas), gastrula (gast), limb bud embryo I (Lbe1), limb bud embryo II (Lbe2), larva in membrane I (Lim1), larva in membrane II (Lim2), nauplius I (N1), nauplius II (N2), nauplius III (N3), nauplius VI (N6), zoea I (Z1), zoea II (Z2), zoea III (Z3), mysis I (M1), mysis II (M2), mysis III (M3) and post-larva 1 (P1).

We also assessed the expression profiles of *LvCXE*s at early developmental stages of *L. vannamei*. Obviously, the expression levels of all *LvCXE*s could be clustered into two modules. Module 1 included *LvCXE4*, *LvCXE6*, *LvCXE9*, *LvCXE10*, *LvCXE11*, *LvCXE13*, *LvCXE15*, *LvCXE16*, *LvCXE19* and *LvCXE21*, in which contained seven GESAG-containing genes. They showed high expressions from zoea I (Z1) to post-larva (P1) stage. In detail, their expressions increased until mysis II (M2) stage and decreased in mysis III (M3) stage, which was the end of metamorphosis. Other *LvCXE*s were clustered in module 2, whose expression levels were relatively low during the whole early developmental stages (Figure 6).

### 2.5. Expression Patterns of LvCXEs during Molting

**Figure 7:**
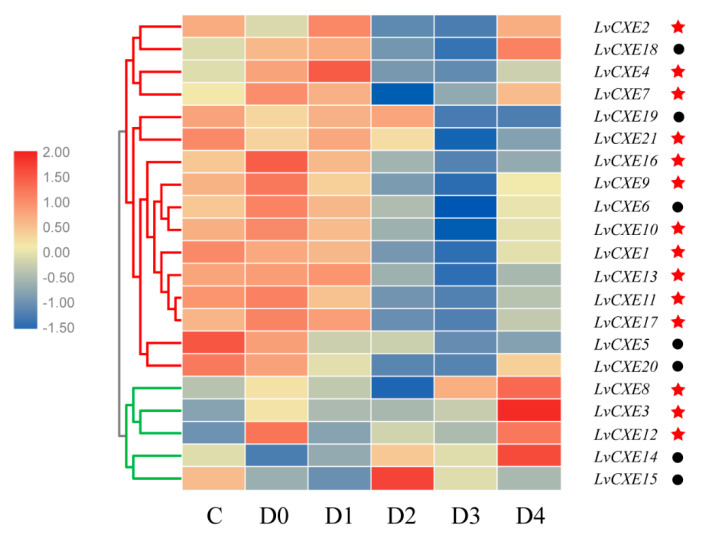
The expression profiles of *LvCXE*s during molt cycles of *L. vannamei*. Red and green clades represente module 1 and module 2, respectively, which were clustered by hierarchical clustering. *LvCXE*s shared similar expression profiles during the molt cycle in each module. The bottom lists different molting stages of *L. vannamei*, from left to right—inter-molt (C) and pre-molt (D0, D1, D2, D3, D4) stage. Red stars represent *LvCXE* containing a GESAG motif and black circles represent *LvCXE*s without a GESAG motif.

All *LvCXE*s were detectable with dynamic expression patterns during the molting cycle. Module 1, including 16 *LvCXE*s, *LvCXE1*, *LvCXE2*, *LvCXE4*, *LvCXE5*, *LvCXE6*, *LvCXE7*, *LvCXE9*, *LvCXE10*, *LvCXE11*, *LvCXE13*, *LvCXE16*, *LvCXE17*, *LvCXE18*, *LvCXE19*, *LvCXE20* and *LvCXE21*, showed similar expression patterns that the expression levels were high at C and D0 stage, decreased in D3 stage, and increased rapidly in D4 stage during the molting period (Figure 7, Supplementary Figure S1). Furthermore, 11 of 14 GESAG-containing *LvCXE*s exhibited molt-dependent expressions, indicating that these expanded *LvCXE*s might be crucial for the molt cycle of *L. vannamei*. However, the results of hierarchical clustering analysis showed that the expression patterns of module 2, including *LvCXE3*, *LvCXE8*, *LvCXE12*, *LvCXE14* and *LvCXE15*, were distinct from module 1. They exhibited low expressions in the C-D3 stage and rapid high expressions in D4 stage.

### 2.6. OTFP Inhibition

**Figure 8:**
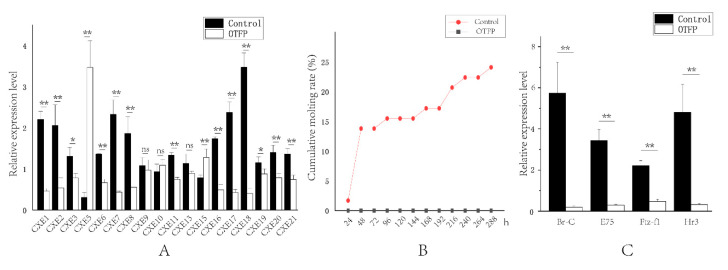
Effect of 3-octylthio-1,1,1-trifluoropropan-2-propanone (OTFP) inhibition on the cumulative molting rate and the expression of *LvCXE*s, *LvBr-C*, *LvE75*, *LvFtz-f1* and *LvHr3*. (**A**). The relative expression levels of *LvCXE*s after OTFP inhibition. (**B**). The cumulative molting rate in control group (red line) and OTFP group (grey line). (**C**). The relative expression levels of *LvBr-C*, *LvE75*, *LvFtz-f1* and *LvHr3* after OTFP inhibition. Significant differences in gene expression levels between two treatments were shown with a star (*) at *p* < 0.05 or two stars (**) at *p* < 0.01. ‘ns’ represents no significant difference.

To further verify the relationship between *LvCXE*s and the molting process of *L. vannamei*, we conducted OTFP inhibition experiments. The real-time PCR results revealed that the expression levels of 13 *LvCXE*s were significantly down-regulated in hepatopancreas after OTFP injection (Figure 8A). The average inhibition efficiency was about 55%, and the highest was 88%. The expression levels of 6 *LvCXE*s showed no significant difference or were not detected. The molting times of each group were recorded and the cumulative molting rate was calculated every 24 h after injection. The results showed that a total of 14 shrimps of control group, accounting for 24.1%, molted in 12 days. However, none of them molted successfully in the OTFP inhibition group. Moreover, eight shrimps died due to their exoskeletons being separated incompletely from the body, which resulted in unsuccessful molting process (Figure 8B). Additionally, many key ecdysone response genes were also affected by OTFP inhibition. The expressions of E75 (ecdysone-induced protein 75), Br-C (Broad-Complex), Hr3 (hormone receptor 3) and Ftz-f1 (Fushi tarazu factor-1) gene were all significantly down-regulated after OTFP inhibition (Figure 8C). Compared with the control group, the relative expressions of E75, Br-C, Hr3 and Ftz-f1 gene in OTFP inhibition group reduced by about 91%, 97%, 93% and 79%, respectively.

## 3. Discussion

Previous studies have showed that a specific expansion of the CXE gene family was found in the *L. vannamei* genome through comparative genomic analysis [36]. Since we found seven, two and zero CXE genes in the genome of *Procambarus virginalis*, *Parhyale hawaiensis* and *Daphnia duplex*, *L. vannamei* has the largest number of CXE genes. The expansion of the gene family is supposed to strengthen the specific biological process or contribute to the adaptive evolution [36,37]. Hence, comprehensive analysis of the CXE gene family would not only study their function systematically, but also illustrate the relationship between its expansion and specific biological characteristics.

The features of sequences determine their biological functions. All LvCXEs identified in this study had conserved motifs and sites similar to those of insect JHEs or other crustacean CXEs. Furthermore, all CXE genes of crustaceans were clustered into a clade of the phylogenetics tree. These results indicated that all LvCXEs belong to the CXE family. Low bootstrap values of some branches may be caused by low sequence similarity between CXEs and JHEs, since there are no large conserved domains. In general, JHE containing a GQSAG motif can degrade JH in insects [38,39]. In this study, only LvCXE5 contained the GQSAG motif, but it was not clustered with insect JHEs in the phylogenetic tree and not expressed in the hepatopancreas or gonads. It indicated that LvCXE5 was not an ortholog of insect JHE. Meanwhile, previous studies found that CXE genes containing the GESAG motif were proposed to have esterase activity for MF in crustaceans [30,32]. Consistently, 14 LvCXEs contained GESAG in our study. Compared with JHE genes containing GQSAG and esterase activity for JH, the difference may be caused by the divergence between crustaceans and insects. Hence, we speculated that LvCXEs containing a GESAG motif may degrade MF in *L. vannamei*. However, since the study on CXE and MF in crustaceans is limited, it is arbitrary to conclude that all LvCXEs are involved in the degradation of MF just according to their sequence characteristics.

To explore the preliminary function of *LvCXE*s, we first analyzed their expressions profiles in various tissues. MF esterase activity has been detected in the hepatopancreas and gonads of many crustaceans [40,41], which were considered to be the main sites for MF inactivation. In the present study, seven *LvCXE*s exhibited high expression in the hepatopancreas, but only *LvCXE17* was expressed in the ovary. This is probably because the shrimp samples of different tissue transcriptomes are not in the stage of gonadal development. Interestingly, MF esterase activity has not been detected in the hemolymph of crustaceans so far [30], but we firstly found that three *LvCXE*s showed a high expression in hemocyte. Additionally, we firstly found that about half of *LvCXE*s were expressed in the antennal gland, which provided clues for the significant role of MF and the antennal gland in osmoregulation [12,42]. Meanwhile, our study also provided clues for better understanding the diverse functions of CXE genes and MF in crustaceans.

Similar to the functions of JH in insects, MF inhibits the metamorphosis of crustaceans [16,17]. Hence, the expression levels of *LvCXE*s were analyzed to further investigate their preliminary function during the developmental stage. Consistent with the increase of JHE activity in the late developmental stage of insects [8,9], 10 *LvCXE*s, including seven GESAG-motif containing genes, began expression in the nauplius, and were highly expressed in the zoeae and mysis stage gradually in *L. vannamei*. These may decrease MF titer and enable shrimp larvae metamorphosis to occur successfully during the early developmental stage. Furthermore, the decreased expression levels of *LvCXE*s at the post-larvae stage may restore MF titer. The above results reveal that *LvCXE*s possess similar function to the JHE gene of insects during early development. However, *L. vannamei* has evolved a pattern that multiple *LvCXE*s control and compensate functionally to maintain normal metamorphosis simultaneously.

The study of the function of *LvCXE*s during the molting cycle is of great significance since MF plays an important role in the molting process. In our study, most *LvCXE*s presented molt-related expressions, suggesting the potential relationship between *LvCXE*s and the molting process of *L. vannamei*. Under normal conditions, the expression levels of *LvCXE*s in module 1 increased significantly at D4 stage, which might result in a rapid decrease of MF titer and lead to the decrease of ecdysone in hemolymph. Similarly, the levels of MF, which fluctuate consistently with ecdysone [20,21,22], rise gradually at the early pre-molt stage but rapidly decline at the late pre-molt stage in the hemolymph of *M. rosenbergii* [43] and *P. clarkia* [15]. However, the injection of OTFP in D3 stage inhibited the transcription of *LvCXE*s and then might lead to the increase of MF titer rapidly, which elevated ecdysone titer in hemolymph abnormally. Furthermore, the synthesis of ecdysone in Y organ was then inhibited owing to the negative feedback, which was supported by the result that the expression levels of transcription factors *LvE75*, *LvBr-C*, *LvHr3* and *LvFtz-f1* were significantly down-regulated. Hence, the above results confirm that the high expression of *LvCXE*s in D4 stage is necessary for the molting cycle, and the CXE gene family plays an important role in the regulation of frequent molting in *L. vannamei*. However, MF exposure or MF injection accelerated the molting process of crustaceans according to previous studies [15,18]. We guess that they did not perform the MF treatment near molting, because they did not check the molting stage of the samples before the experiment. Hence, we speculate that MF can accelerate the molting process at the inter-molt or early pre-molt stage, but hinder the molting process near molting. Furthermore, it is also of concern that the expression patterns of module 2, including *LvCXE3*, *LvCXE8*, *LvCXE12 LvCXE14* and *LvCXE15*, were different from module 1. Besides, the expression levels of *LvCXE3*, *LvCXE8*, *LvCXE12* and *LvCXE14* were very low not only in the hepatopancreas, but also at the early development stages. The above results showed that the function of these five *LvCXE*s should be different or divergent after the gene family expansion. However, there is no significant difference in the amino acid composition of their domains and key sites. It is speculated that the differences in their expression patterns are caused by different promoter regions. Of course, it needs more evidence to support this speculation.

## 4. Materials and Methods

### 4.1. Genome and Transcriptome Data Resources

The genomic data of *L. vannamei* were obtained from our laboratory (NCBI GenBank, PRJNA438564) [36]. Our previous research conducted RNA-seq on various libraries. Five larval stages, including embryo, nauplius, zoea, mysis and post-larvae [44]; eight molting stages, including inter-molt (C), pre-molt (D0, D1, D2, D3, D4) and post-molt (P1 and P2) stages [45]; and 16 adult tissues, including antennal gland, brain, hemocyte, epidermis, eyestalk, gill, hepatopancreas, heart, intestine, abdominal muscle, lymphoid organ, ovary, stomach, testis, thoracic ganglion and abdominal ganglion [46].

### 4.2. The Characterization of CXE Gene Family in L. vannamei

The CXE protein sequences of the morotoge shrimp *Pandalopsis japonica* (F5A5Q6), Chinese mitten crab *Eriocheir sinensis* (A0A1L5JHT6) and *Drosophila melanogaster*-JHE (NP_001286476.1) were used as query to search against all unigene datasets by tblastn (E-value ≤ 10^−6^) and the whole genome predicted proteins by blastp (E-value ≤ 10^−6^). Then, redundant sequences were removed using the CAP3 program [47]. The candidate sequences were submitted to ORF Finder (https://www.ncbi.nlm.nih.gov/orffinder/) and the ExPASy translate tool (http://web.expasy.org/translate/) to obtain deduced amino acid sequences. The integrity of the carboxylesterase domain was predicted using SMART (http://smart.embl-heidelberg.de/) and InterPro database (http://www.ebi.ac.uk/interpro/) (E-value ≤ 10^−50^). The presence of a signal peptide was analyzed by SignalP 4.1 server (www.cbs.dtu.dk/services/SignalP/). A multiple alignment was performed using ClustalW [48] and visualized by jalview [49]. *LvCXE*s were identified if they contained the following three structures—(1) a putative signal peptide in N-terminus (apart from incomplete sequences), (2) conserved catalytic triad (Ser-Glu-His) residues, and (3) a CXE-specific GxSxG motif. The theoretical isoelectric point (pI) and molecular weight (Mw) were calculated using online tool (http://web.expasy.org/compute_pi/). The conserved motifs were analyzed using the MEME program (http://meme-suite.org/) with default parameters [50] and visualized by TBtools [51]. To determine the locations of *LvCXE*s in *L. vannamei* genome, all *LvCXE* sequences were mapped to the whole genome scaffolds by blastn program (E-value ≤ 10^−30^). The exon/intron positions were then determined when the identity of aligned region > 97%. The exon/intron boundaries were manually checked and the structures were finally visualized with the Gene Structure Display Server web tool (http:/gsds.cbi.pku.edu.cn/).

### 4.3. Phylogenetic Analysis

The full-length amino acid sequences of CXE from crustaceans and JHE from insects were collected from GenBank databases. All collected sequences were aligned using MUSCLE program (v3.8.31) [52] with default parameters. Then the best-fit model for constructing the phylogenetic tree was selected by ProtTest 3.4.2 (parameter: -all-distributions -F -AIC -BIC -tc 0.5) [53]. A maximum likelihood (ML) phylogenetic tree of LvCXEs was generated by RAxML 8.0.26 [54] with a WAG+I+G+F model. Meanwhile, the ML phylogenetic tree of arthropod JHEs/CXEs was generated with a WAG+G+F model. All trees were tested with 100 bootstrap replicates and finally visualized using iTOL [55].

### 4.4. Expression Profiles

In previous studies, the RPKM (reads per kilobases per million reads) values of all transcripts of 20 early development stages, 8 molting stages and 16 adult tissues were calculated [44,45,46]. The RPKM values of all *LvCXE*s were extracted and then normalized with log2 conversion. Heat maps were then created and clustered with hierarchical clustering using TBtools software [51].

### 4.5. OTFP Inhibition

To investigate the relationships between *LvCXE*s and the molting process of *L. vannamei*, a total of 120 shrimps (about 5 cm for body length) in D0-D4 stage were selected and maintained in filtered seawater at a temperature of 25 ± 1 °C with continuous aeration. Before the experiment, all shrimps were kept in the aquarium for 3 days to acclimate them to the laboratory conditions. They were then randomly selected into a control group and experimental group. For each group, three replicates were conducted. The control group was injected with 10 µL mixture of ethanol and PBS (volume ratio 3:7) and the experimental group was injected with 10 µL 4.19 × 10^−3^ mol/L OTFP solution, which was diluted by the mixture of ethanol and PBS (volume ratio 3:7). The molting times of each group were recorded and the cumulative molting rate was calculated every 24 h after injection. To further quantify the expression levels of some molt-related genes, 20 shrimps in D3-D4 stage were randomly selected into a control group and experimental group. The control group was injected with 10 µL mixture of ethanol and PBS (volume ratio 3:7) and the experimental group was injected with 10 µL 4.19 × 10^−3^ mol/L OTFP solution, which was diluted by the mixture of ethanol and PBS (volume ratio 3:7). At 48 h postinjection, the hepatopancreas of four shrimps from each group was collected separately for RNA extraction.

### 4.6. RNA-Isolation and qRT-PCR

Total RNA was extracted using RNAiso Plus reagent (TaKaRa, Kyoto, Japan) according to the manufacturer’s instructions. Agarose electrophoresis and NanoDrop 2000 (Thermo Fisher Scientific, Waltham, MA, USA) were used to detect the quality and the concentration of RNA, respectively. First-strand cDNA was synthesized using 1 µg of total RNA with a PrimeScript RT reagent kit (TaKaRa). According to the instruction, the mixed primer included Oligo d(T) primer and random hexamer primer was used and the genomic DNA had been removed by DNase treatment for cDNA synthesis. cDNA was synthesized according to the following procedure—37 °C for 1 h, and 85 °C for 5 s. The cDNA samples were stored at −80 °C for further use.

SYBR Green-based quantitative real-time PCR (qRT-PCR) was performed to detect the expression levels of *LvCXE*s and four ecdysone response genes in the hepatopancreas using THUNDERBIRD^TM^ SYBR^®^ (TOYOBO, Osaka, Japan). Four technical replicates were conducted for each gene to eliminate the systematic errors. The 18S rRNA was used as an internal standard. Primer sequences were listed in Supplementary Table S1. qPCR was performed with an Eppendorf Mastercycler ep realplex (Eppendorf, Hamburg, Germany) using the following program—denaturation at 95 °C for 15 min; 40 cycles of 95 °C for 15 s, annealing temperature for 15 s, and 72 °C for 20 s. The PCR melting-curve was used to check the specificity of the PCR product. In addition, relative expression levels were calculated with the formula 2^−ΔΔC^_T_ using the comparative *C*_T_ method [56]. An unpaired two-tailed t test was used for statistical analysis by R (version 3.3.3). *p* value < 0.05 was considered statistically significant.

## 5. Conclusions

In summary, a total of 21 complete *LvCXE*s were identified in the *L. vannamei* genome. Their genome localizations, gene structures and conserved domains were comprehensively analyzed. The CXE genes with a GESAG motif expanded specifically in the *L. vannamei* genome, which increased the diversity of gene structures and functions. At the end of the early developmental stage, the high expression levels of *LvCXE*s may enable shrimp larvae to develop successfully. The high expression levels of *LvCXE*s in D4 stage are essential for the molting cycle. In contrast, the abnormal function of *LvCXE*s will lead to the failure of the molting process. In conclusion, the expansion of the CXE gene family is related to precise regulation for early development and frequent molting in *L. vannamei*.

## Figures and Tables

**Table 1:** Characteristics of 21 *LvCXE*s identified in penaeid shrimp genome.

Gene ID	Amino Acids	Mw (kD)	pI	Location
*LvCXE1*	564	61.4	5.07	LVANscaffold_866: 18624–23041
*LvCXE2*	488	54.3	6.55	LVANscaffold_883: 576355–581238
*LvCXE3*	574	64.0	5.69	LVANscaffold_883: 575275–603536
*LvCXE4*	577	64.7	5.58	LVANscaffold_962: 831167–839422
*LvCXE5*	575	63.5	6.03	LVANscaffold_962: 839832–855606
*LvCXE6*	544	60.0	4.78	LVANscaffold_1108: 73812–101034
*LvCXE7*	569	63.2	4.88	LVANscaffold_1123: 580276–596930
*LvCXE8*	585	64.3	5.79	LVANscaffold_1168: 597704–610937
*LvCXE9*	581	65.1	5.34	LVANscaffold_1839: 157651–172084
*LvCXE10*	573	63.3	5.13	LVANscaffold_1839: 184142–188709
*LvCXE11*	585	64.8	5.60	LVANscaffold_1907: 1025753–1038397
*LvCXE12*	>605	66.6	5.75	LVANscaffold_2316: 553488–572600
*LvCXE13*	669	74.5	4.95	LVANscaffold_2381: 397192–408701
*LvCXE14*	572	64.1	8.98	LVANscaffold_2639: 313127–321369
*LvCXE15*	561	62.1	5.77	LVANscaffold_2654: 959807–971335
*LvCXE16*	565	62.2	4.97	LVANscaffold_2937: 352862–369967
*LvCXE17*	621	69.8	4.53	LVANscaffold_2938: 299225–309403
*LvCXE18*	627	68.1	6.45	LVANscaffold_3071: 91750–98940
*LvCXE19*	561	62.1	6.40	LVANscaffold_3486: 45723–66803
*LvCXE20*	592	65.7	6.10	LVANscaffold_199: 131030–134929
*LvCXE21*	583	65.3	6.08	LVANscaffold_2382: 54122–66300

**Table 2:** The conserved motif organization of LvCXEs.

Gene ID	Signal Peptide	RF	DQ	GxSxG	E/D	H
*LvCXE1*	+	RL	DQ	GESAG	D	H
*LvCXE2*	+	RL	DQ	GESAG	E	/
*LvCXE3*	+	RL	DQ	GESAG	D	H
*LvCXE4*	+	RF	DQ	GESAG	E	H
*LvCXE5*	+	RL	DQ	GQSAG	E	H
*LvCXE6*	+	RF	DQ	GGSAG	E	H
*LvCXE7*	+	RF	DQ	GESAG	D	H
*LvCXE8*	+	RL	DQ	GESAG	E	H
*LvCXE9*	+	KL	DQ	GESAG	E	H
*LvCXE10*	+	RL	DQ	GESAG	D	H
*LvCXE11*	+	RF	DQ	GESAG	E	H
*LvCXE12*	−	RF	DQ	GESAG	E	H
*LvCXE13*	+	RF	DQ	GESAG	E	H
*LvCXE14*	+	RF	DQ	GLSAG	E	H
*LvCXE15*	+	RF	DQ	GVSAG	E	H
*LvCXE16*	+	RF	DQ	GESAG	E	H
*LvCXE17*	+	RL	DQ	GESAG	E	H
*LvCXE18*	+	RW	DQ	GFSAG	E	H
*LvCXE19*	+	RF	DQ	GVSSG	E	H
*LvCXE20*	+	RF	DQ	GISAG	D	H
*LvCXE21*	+	RF	DQ	GESAG	E	H

## Supplementary Materials

Supplementary Materials can be found at https://www.mdpi.com/1422-0067/21/15/5444/s1. Figure S1: The normalized expressions of LvCXEs in module 1 of Figure 7. Each line represents the expression level of a LvCXE in module 1. Table S1: Primers for real-time qRT-PCR.
