# Supplementary material for: Genomic Characterization and Expression of Juvenile Hormone Esterase-Like Carboxylesterase Genes in Pacific White Shrimp, Litopenaeus vannamei
Source: Int J Mol Sci. 2020 Jul 30;21(15):5444. doi: 10.3390/ijms21155444 (PMC7432913; doi:10.3390/ijms21155444)
Supplement: Supplementary file 1 [file ijms-21-05444-s001.pdf]

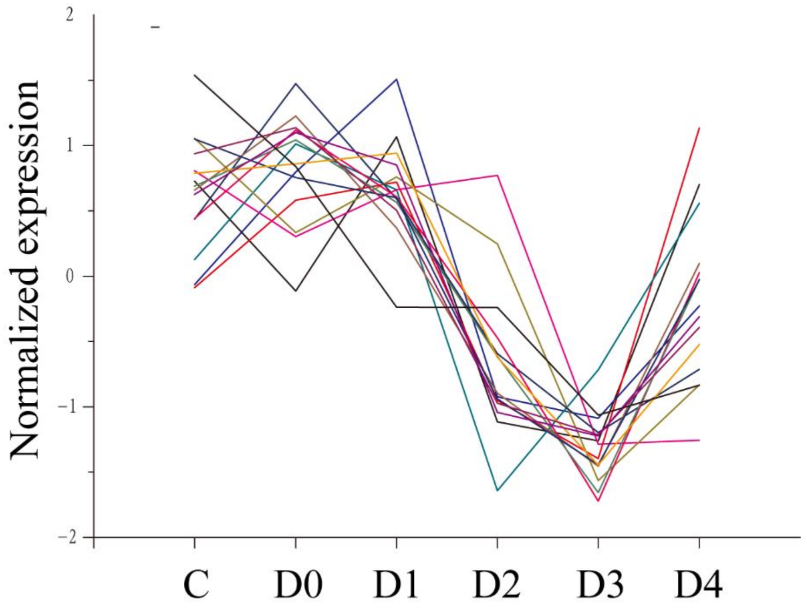

Supplementary Figure S1. The normalized expressions of *LvCXE*s in module 1 of Figure 7. Each line represents the expression level of a *LvCXE* in module 1.

Supplementary Table S1. Primers for real-time qRT-PCR

Gene	Forward primer (5′-3′)	Reverse primer (5′-3′)
18S	TATACGCTAGTGGAGCTGGAA	GGGGAGGTAGTGACGAAAAAT
LvCXE1	CGGAGCCATCGTAACACAAC	GAAGGTCAAGTCGCCAAGATAG
LvCXE2	CGCTCTGCCTCTTGTTGATG	TCCTTCGTCTCTTGTGATTCCA
LvCXE3	AATGCTCGTCGTTCTTGTAACT	GATGCCGTAGTAGGAGTGGAA
LvCXE4	ATCAGTGGCGACAGGATAGTT	AGGAGAGGAAGAGGCGAGTA
LvCXE5	TTGCCTGTAATGGTGTTCTTCC	CTGGTCCTTCAAGCCGTAGT
LvCXE6	GCGTCAGTCCACTACCAGAT	AACCGAGCGGCGATATTCT
LvCXE7	CGATGACGAGTGCCAGTGA	GGTGAAGCGGTCCTTGTAGA
LvCXE8	CGAACCCATTGACCTTCCTTAC	CTTCTGAGCAACCGTCCTTATG
LvCXE9	GCAACTCTCCTTCGGTGACA	GTCCACATGGCGGTGATGA
LvCXE10	CAGTATGATGGAGCCGATGGT	GTCGTCAGGTCGTGTGGAA
LvCXE11	CCGAATACCTGGCGAGAAGA	AGGTGGCACATTCCGAACA
LvCXE12	AGGTCGCTGTGATGGTCTG	CAAGAGTGCCGAGTCTGAAC
LvCXE13	CGAGGCACAAGAAGATTGACAT	AGATGAGCAGCGTCTGGTTC
LvCXE14	ATCATACGACTGGCGATATTCAC	AGCATACGGAATACTCCTGAAGA
LvCXE15	ATTCCTCTCCAGTGAAGATGCT	CCGCCAAGGTTCCTGATGT
LvCXE16	TAATGAAGAGGTCCGCCACTT	ATCCTACAACAGCAGAGTCCTT

LvCXE17	TGCGACGACCTCTTCTACC	CAAGATGACTTCTCTGAGTGTGA
LvCXE18	ACGAAGACTGTCTCCATCTGAA	GTAGTTGACGGTGACGAGGA
LvCXE19	CCACAGGCGATCTCAGGTT	ACATTCAGGTAGAGGCAGTCTT
LvCXE20	GAAGGCTCGCAGAAGGTTC	GGCTCGTCAGGAAGGAAGT
LvCXE21	GGTGGAGATGTGGACGAACT	GAGGAAGTGATGGCTAAGTAGGA
LvE75	GATACATTCAGGCTTGGGTGC	TGTCCGCCTGAGAGTGAGAATA
LvBr-c	CCCCCACACTCCTGTACTAA	TAACCTTGGTATCCTGCGG
LvFtz-f1	ACACACATTAGTACGGGGG	CATTAGAGTCGGGGTCAAC
LvHr3	GGTTAGTATGAACTGGCACAAT	ATCAAGCCCATCATTACGTT
